# A biochar selection method for remediating heavy metal contaminated mine tailings

**DOI:** 10.1007/s13762-024-05621-9

**Published:** 2024-04-15

**Authors:** J. A. Ippolito, T. F. Ducey, K. A. Spokas, K. M. Trippe, M. G. Johnson

**Affiliations:** 1 School of Environment and Natural Resources, The Ohio State University, Columbus, OH 43210, USA; 2 Agricultural Research Service, Coastal Plains Soil, Water, and Plant Research Center, United States Department of Agriculture, Florence, SC 29501-1242, USA; 3 Soil and Water Management Unit, Agricultural Research Service, United States Department of Agriculture, St. Paul, MN 55108-6028, USA; 4 Forage Seed and Cereal Research Unit, Agricultural Research Service, United States Department of Agriculture, Corvallis, OR 97331-8539, USA; 5 Pacific Ecological Systems Division, Center for Public Health and Environmental Assessment, U.S. Environmental Protection Agency, Corvallis, OR 97333-4902, USA

**Keywords:** Biochars, Heavy metal bioavailability, Heavy metal sorption, Synthetic precipitation leaching procedure

## Abstract

Approximately 390,000 abandoned mines across the US pose considerable, pervasive risks to human and environmental health; world-wide the problem is even greater. Lime, organic materials, and other amendments have been used to decrease metal bioavailability (e.g., Cd, Cu, Mn, Ni, Zn) in contaminated mine wastes and to promote plant community establishment for tailings stabilization. Biochar properties (e.g., alkaline pH, metal sorbing capabilities, available nutrients, improved soil water retention) make it a potential amendment for remediating metal contaminated mine tailings. A three-step procedure was developed to identify biochars that were most effective at reducing heavy metal availability, retaining metals, and subsequently selecting biochars for use in a soil amendment laboratory trial to ultimately be utilized in heavy metal contaminated mine land settings: Step (1) a synthetic precipitation leaching procedure extract of mine tailings was produced, representing potentially available metals, and used to identify metal removal properties of 28 different biochars (e.g., made from various feedstocks and pyrolysis or gasification conditions); Step (2) evaluate how well biochars retained previously sorbed metals; and Step (3) laboratory evaluation of the most promising biochars that removed and did not releases metals, applied at 0, 1, 2.5, and 5% (by wt) to mine tailings for reducing metal bioavailability. The reported methodology and results from this study could be used to quickly identify specific biochars and application rates to reduce mine tailings metal availability and aid in future remediation of abandoned mine sites globally.

## Introduction

The United States contains more than 390,000 abandoned mines (GUS GAO 2020). In the past, these were areas where ore extraction, beneficiation, and processing occurred for the recovery of precious metals (e.g., Au, Ag, Cu, Zn). Some of these sites generate acid mine drainage; ~ 33,000 of these types of sites are found in the western US alone (Mittal 2011). Acid mine drainage can lead to greater environmental heavy metal bioavailability (e.g., Cd, Cu, Mn, Ni, Zn), and in turn, tailings (i.e., earthen materials remaining after precious metal extraction), soil, surface water, and ground water degradation (Ippolito et al. 2017; Ramontja et al. 2011). To reduce acid-generation and metal bioavailability in mine-impacted locations, products such as lime and organic amendments have been successfully implemented and led to improvements in plant growth and reclamation success at these sites (Brown et al. 2005, 2007; Rieder et al. 2013). However, other environmentally-friendly products created from relatively local sources, that could sequester heavy metals while addressing climate change issues via enhanced long-term soil C sequestration, may also be utilized. These products are associated with the conversion of waste biomass into biochars.

Biochar has been proven to decrease heavy metal bioavailability within contaminated soils. For example, Cui et al. (2011) added increasing amounts of wheat straw biochar (up to 40 Mg ha^−1^) to a Cd contaminated rice paddy soil. The authors noted that increasing biochar rates increased soil pH, decreased soil Cd bioavailability by up to 52%, and reduced Cd within rice by up to 62%. Reductions were likely due to biochar causing Cd surface complexation or the formation of insoluble Cd mineral phases (Yin et al. 2017). Similarly, biochar applications have been shown to decrease Cu in contaminated soils, due to a rise in pH the subsequent formation of Cu as organically complexed or insoluble oxyhydroxide and carbonate mineral phases (Ippolito et al. 2017; Meier et al. 2017; Rodriquez-Vila et al. 2017). Kumar et al. (2018) added cattle manure biochar (up to 5% by wt) to a soil spiked with Zn and aged for 60 days. The authors noted that as biochar rate increased, Zn content in *Ficus elastica* leaves decreased, lower Zn concentrations were leached, and that biochar particles acted as a sink for Zn. Xu et al. (2017) noted a similar Zn response with increasing biochar application rates (up to 2% by wt) applied to a contaminated Zn soil. The above results suggest that biochar may be an excellent choice for reducing heavy metal bioavailability in metal-contaminated soils and tailings.

However, there are also published literature results suggesting that no metal availability change occurs, or a negative response occurs, when biochar is added to metal contaminated soil. Peltz and Harley (2016) added 30% biochar (by volume) to a Zn contaminated soil. Their two-year trial showed little change in extractable, leachable Zn concentrations as compared to a control. Kelly et al. (2014) added pine wood biochar (up to 30% by volume) to a Zn contaminated mine tailing and performed repeated leachings over a two-month period of time. The authors observed a significant increase in Zn leachate concentrations with increasing biochar applications. Ippolito et al. (2019) added a fast pyrolysis hardwood biochar (up to 2% by wt) to soils spiked with Cu (up to 1000 mg kg^−1^) to study the effect on alfalfa growth, Cu uptake, and reductions in available soil Cu concentrations. Increasing biochar application rates had no effect on alfalfa yield, did decrease plant Cu concentrations, but had no effect on available soil Cu concentrations.

Existing literature is certainly contradictory, with the bulk of previous studies explaining biochar sorption/desorption effects on single elements. This may be due to the differences in the biochars used in these studies because biochar properties are a function of feedstock, pyrolysis conditions and pre- and post-pyrolysis treatments. Additionally, the sorption of heavy metals by biochar may vary in the presence of multiple elements due to competition for binding sites (Natasha et al. 2021); this will likely be encountered in heavy metal contaminated mining impacted soils/tailings. Therefore, a laboratory screening approach would be useful for identifying biochars that would be best suited for reducing soil or tailings heavy metal availability prior to setting up a full-fledged, on-site reclamation/revegetation program. The objectives of the current study were to develop a simple screening tool whereby biochar users and applicators could objectively identify biochars that are most effective at remediating heavy metal contaminated soils or tailings in terms of sequestering and not releasing heavy metals back into a bioavailable form. Ultimately, this work may provide biochar utilizers with a more practical approach of utilizing biochars in heavy metal contaminated settings, ultimately improving plant growth and overall reclamation success.

## Materials and methods

### Biochars

Twenty-eight biochars, made from 13 different feedstocks, were utilized; granulated activated charcoal (GAC), made from coconut shells, was included for comparative purposes. All feedstocks were oven-dried at 60 °C, then crushed or ground to pass a 4-mm sieve; sieved feedstocks were used to create biochars. Most biochars were created using a 4-h hold time at pyrolysis temperatures of 300, 500, or 70 °C. One additional biochar, made via gasification (1100 and 1400 °C) of mixed conifer logging slash, was provided by the USDA-Agricultural Research Service. Table 1 presents these biochars along with their associated acronyms.

### Metal contaminated mine tailings collection

**Fig. 1:**
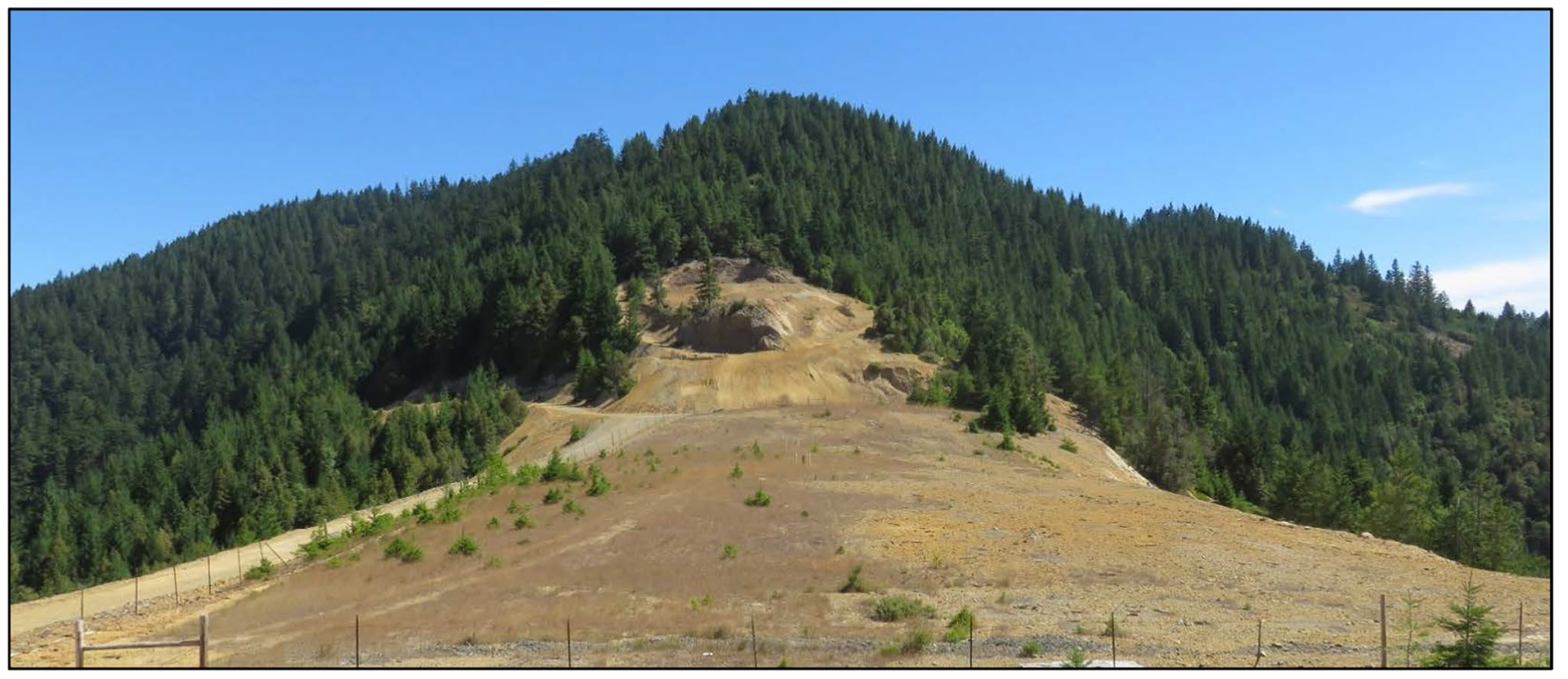
An eastward view of the aboveground remains at the abandoned Formosa Mine site near Riddle, Oregon, USA. A large encapsulation mound (foreground) was a repository for low-grade waste ore. Upslope is the proposed target area for tailings amendments (e.g., biochar) and remediation. Mine tailings were collected in the upslope area for the current study.

The abandoned Formosa Mine, located approximately 16 km south of Riddle, Oregon, USA (1000–1200 m in elevation), is on the US EPA Superfund National Priorities List because heavy metals and acid mine drainage pose a risk to human health and the environment. The 31 ha Formosa mine was originally mined for Cu and Zn from approximately 1910–1937. The mine was reopened in 1989 with mining operations ceasing in 1993. Belowground mining works were filled with mine tailings, crushed ore, and limestone. Following some surface reclamation, the mining companies left the area. The primary aboveground mine disturbance encompasses approximately 10 ha. A site picture is presented in Fig. 1, with mine tailings for this study collected in the upslope area, an area targeted for near-future reclamation and revegetation. The tailings were collected from the top 15 cm (pH ≈ 2.7), returned to the lab, air-dried, passed through a 2-mm sieve, and then used for subsequent analyses.

### Synthetic precipitation leaching procedure (SPLP)

The EPA Method 1312 SPLP (USA EPA 1994) was followed to create a metals-containing extract of the Formosa Mine tailings, with the SPLP extract considered to mimic metals that may be leachable or in the tailings solution and plant-available. The SPLP solution had a pH of 4.2, created from the incremental addition of a 60:40 mix of concentrated H_2_SO_4_ and HNO_3_ to milli-Q H_2_O. The SPLP solution was mixed with sieved, Formosa Mine tailings at a 1:20 solid:solution ratio (50 g of 2-mm sieved tailings:1000 mL SPLP solution) on an end-over-end mixer for 24 h. After 24 h, the solution was passed through a 0.45 μm filter.

#### Step 1: heavy metal sorption—reacting SPLP solution with biochar

In triplicate, 0.25 g of biochars were placed into 50 mL centrifuge tubes and shaken with 25 mL of SPLP extract (1:100 solid:solution ratio) for 24 h at 25 °C. A 24 h shaking period was utilized as this shaking time is common for biochar-heavy metal sorption studies (e.g., Park et al. 2016; Zhao et al. 2019; Islam et al. 2021; Carnier et al. 2022). Afterwards, solutions were separated from the biochars using a 0.45 μm filter in a column vacuum filter apparatus. Solutions were analyzed for Cd, Cu, Mn, Ni, and Zn, the five dominant metals present in the Formosa Mine tailings, via inductively coupled plasma-optical emission spectroscopy (ICP-OES). The biochars collected on the filters were washed three times with Milli-Q water, allowed to air-dry and then utilized to determine if the sorbed heavy metals could be desorbed from the biochars.

#### Step 2: heavy metal desorption from biochar

In triplicate, 0.15 g of air-dried biochars (from the step above) were placed into 50 mL centrifuge tubes and shaken with 15 mL of 0.01 M CaCl_2_ (1:100 solid:solution ratio) for 24 h at 25 °C. As with the sorption study, a 24 h shaking period was used as this shaking time is common for biochar-heavy metal desorption studies (e.g., Bogusz et al. 2017; Carnier et al. 2022). The 0.01 M CaCl_2_ extraction is regarded as a measure of trace metal bioavailability (Ippolito et al. 2017; Pueyo et al. 2004). Afterwards, the solution was separated from the biochar using a 0.45 μm filter in a vacuum filter apparatus. The filtrate was analyzed for Cd, Cu, Mn, Ni, and Zn via ICP-OES.

#### Step 3: laboratory analyses of most effective for mine tailings heavy metal sorption

Biochars that reduced heavy metal concentrations within the SPLP extract, followed by those biochars that did not rerelease metals back into solution based on the 0.01 M CaCl_2_ extraction, were utilized in a biochar-Formosa Mine tailings experiment. In quadruplicate 50 mL centrifuge tubes, sieved Formosa mine tailings was combined with 0, 1, 2, or 5% of each biochar (by wt), to a total weight equal to 3.00 g. Next, 30 mL of 0.01 M CaCl_2_ was added, the mixtures were shaken for 2 h at 25 °C, filtered through a 0.45 μm filter, and analyzed for Cd, Cu, Mn, Ni, and Zn via ICP-OES. This shaking period has been correspondingly related to potential reductions in heavy metal bioavailability (Ippolito et al. 2017; Pueyo et al. 2004).

### Statistical analyses

Analysis of variance was performed on all data using the Proc GLM model in SAS version 9.4 (SAS Institute 2012) at a *p* < 0.05 significance level. When significance was present, a Tukey’s range test was used to identify differences between biochars for heavy metal sorption from the SPLP solution, desorption in the presence of 0.01 M CaCl_2_, and heavy metal sorption in the presence of Formosa Mine tailings.

## Results and discussion

Cadmium, Cu, Mn, Ni, and Zn were the most abundant heavy metals present in the Formosa Mine tailings SPLP extract, averaging 59, 260, 630, 16, and 8444 mg L^−1^, respectively. These concentrations were the initial point by which biochar metal removal was judged.

### Step 1: heavy metal sorption—reacting SPLP solution with biochar

**Fig. 2:**
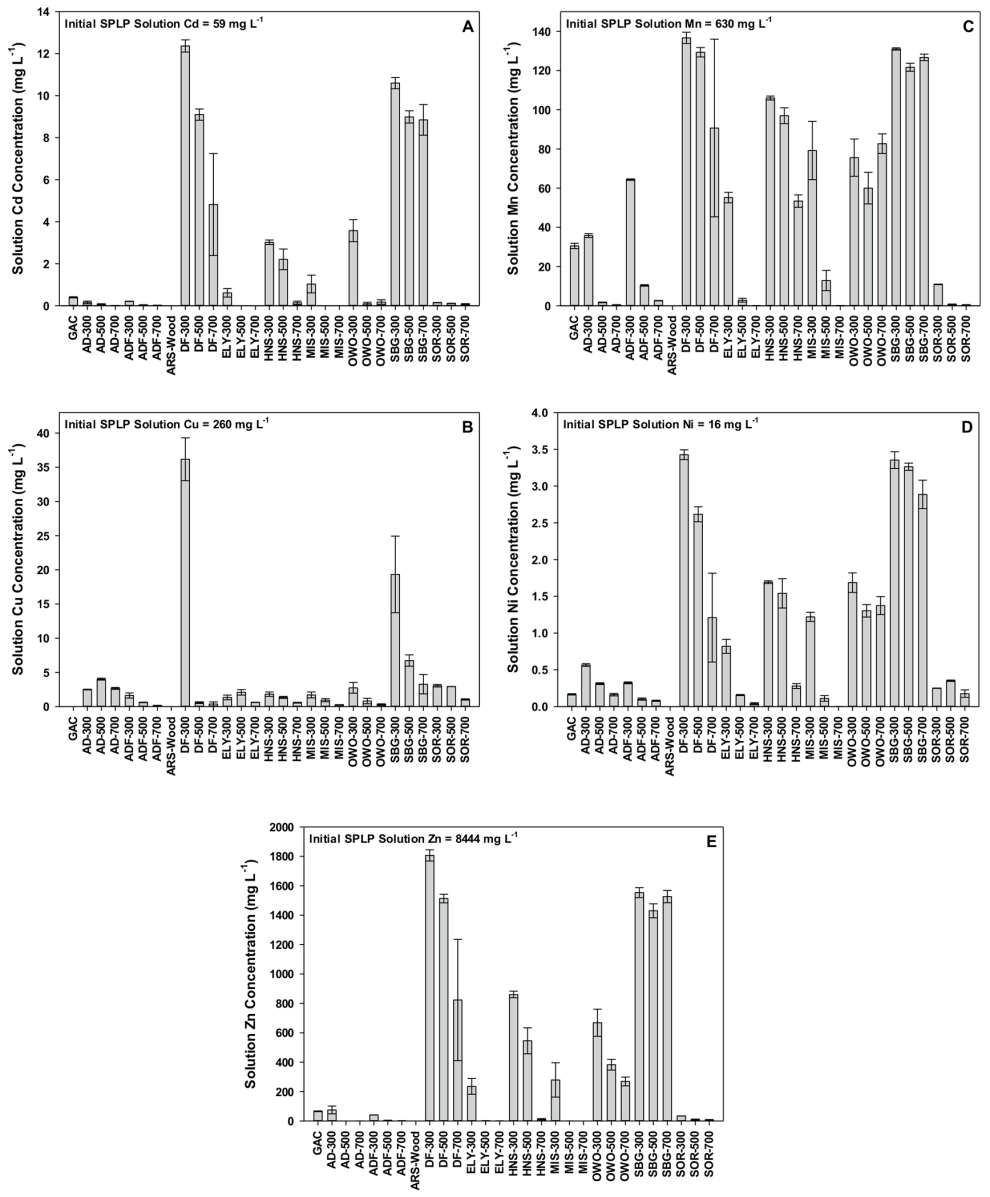
The amount of SPLP Cd (**A**), Cu (**B**), Mn (**C**), Ni (**D**), and Zn (**E**) remaining in solution after reacting granulated activated C (GAC) and various biochars (acronyms located in Table 1) with a synthetic precipitation leaching procedure extract containing metals from Formosa Mine tailings. Error bars represent the standard error of the mean.

The amount of Cd, Cu, Mn, Ni, and Zn remaining in solution, after reacting the SPLP extract with GAC or various biochars, is presented in Fig. 2A–E. Data is presented in mg L^−1^ for comparative purposes to the initial metal concentration in the SPLP solution. The suite of biochars reduced: (a) Cd from 59 mg L^−1^ to between 0 and 12 mg L^−1^; (b) Cu from 260 mg L^−1^ to between 0 and 35 mg L^−1^; (c) Mn from 630 mg L^−1^ to between 0 and 138 mg L^−1^; (d) Ni from 16 mg L^−1^ to between 0 and 3.5 mg L^−1^; and (e) Zn from 8444 mg L^−1^ to between 0 and 1800 mg L^−1^. Although specific sorption phenomena were not examined in the current study, biochars have been known to sorb heavy metals via complexation with organic functional groups, electrostatic attraction to π-electrons, and mineral precipitation (e.g., Ippolito et al. 2013, 2019; He et al. 2019; Joseph et al. 2021). Furthermore, this data suggests the apparent lack of competitive sorption occurring for the concentration ranges examined, likely due to the variety of aforementioned heavy metal complexation reaction sites present within biochars studied.

A summary of the percent removal of each metal, as a function of the initial SPLP metal concentration, is presented in Table 2. For each of these metals, a range of removal existed depending on the specific biochar (e.g., Cd = 80–100%; Cu = 86–100%; Mn = 78–100%; Ni = 79–100%; Zn = 79–100%). Out of all the biochars, there was > 95% SPLP metals removal for the following elements: Cd = 20 biochars; Cu = 26 biochars; Mn = 12 biochars; Ni = 16 biochars; and Zn = 19 biochars. Data in Table 2 also suggests that 16 biochars and the GAC acted similarly in terms of removing an equal amount of all heavy metals from the SPLP extract solution. One might consider ARS-Wood, MIS-700, ELY-700, ADF-700, SOR-700, ELY-500, AD-700, ADF-500, MIS-500, SOR-500, AD-500, SOR-300, HNS-700, AD-300, ADF-300, and ELY-300 biochars, and GAC for heavy metal contaminated mine land reclamation. However, it is recommended, in an additional step, to identify whether biochar-sorbed metals are readily desorbed. If readily desorbed, this would defeat the purpose of biochar use for heavy metal contaminated mine land reclamation.

### Step 2: heavy metal desorption from biochar

The amount of Cd, Cu, Mn, Ni, and Zn desorbed, after reacting biochars (from Step 1) for 24 h in 0.01 M CaCl_2_ (i.e., an indicator of bioavailability; Ippolito et al. 2017), is presented in Table 3. This step can be considered a measure of sorbed metal retention. Considering the total amount of heavy metals desorbed, the previous 16 biochar candidates and GAC (from Step 1) were reduced to 10 biochars, all of which performed statistically better than GAC in terms of retaining metals. ARS-Wood, AD-700, ADF-300, ADF-700, ELY-500, ELY-700, MIS-700, SOR-300, SOR-500, and SOR-700 biochars released the least amount of metals. These biochars could be considered the most effective candidates for use in heavy metal binding lab, greenhouse, and potentially field experiments, by mixing into heavy metal contaminated Formosa mine tailings.

As part of a larger project in Poland, Bogusz et al. (2017) performed a biochar-metal spiking experiment using Cd, Cu, Ni, and Zn. Following a heavy metal sorption step, heavy metal desorption was attempted. The authors noted that ~ 3%, 6%, 8%, and 1.3% of sorbed Cd, Cu, Ni, and Zn was desorbed, respectively. Bogusz et al. (2017) observed the greatest desorption with Ni, likely due to biochar surface complexation (Uchimiya et al. 2011), a weak bond that is easily broken. In the current study, however, Ni was not desorbed from almost all biochars, suggesting that other sorption mechanisms were at play (e.g., interaction with *«*-electrons, sorption onto mineral surfaces, or mineral precipitation). Sorption mechanisms for Cd, Cu, and Mn must have followed phenomena similar to Ni, as little of each metal was desorbed. Zinc desorption varied, depending on biochar type.

### Step 3: laboratory analyses of most effective for mine tailings heavy metal sorption

Based on data in Table 3, out of the most effective biochars for retaining heavy metals, the top three biochars (SOR-700, SOR-500, and ARS-Wood) were chosen for a laboratory study with Formosa mine tailings. Three other biochars (MIS-700, ADF-700, AD-700) were also chosen as they represented the range of least heavy metals desorbed. All biochars were mixed into Formosa mine tailings at rates of 0, 1, 2.5, and 5% (by wt), shaken for two hours in 0.01 M CaCl_2_ solution (i.e., bioavailable metals), filtered, and analyzed for Cd, Cu, Mn, Ni, and Zn concentrations.

**Fig. 3:**
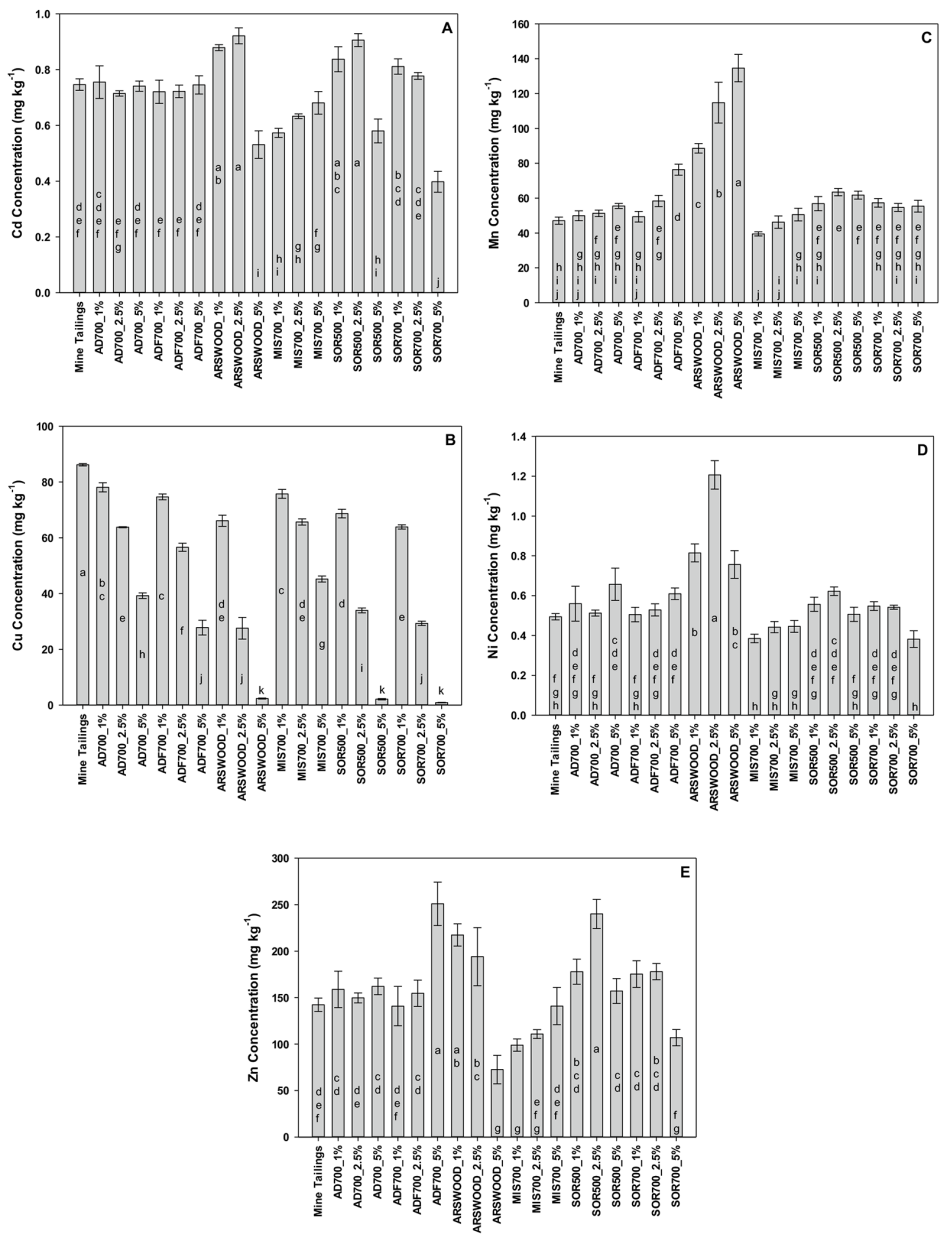
The amount of 0.01 M CaCl_2_ extractable Formosa Mine tailings Cd (**A**), Cu (**B**), Mn (**C**), Ni (**D**), and Zn (**E**) after 2 h shaking with increasing amounts (by wt) of various selected, most effective biochars (based on heavy metal sorption/desorption observations; acronyms located in Table 1). Error bars represent the standard error of the mean.

Results of Formosa Mine tailings heavy metal sorption by the selected biochars are presented in Fig. 3A–E. All bioavailable Cd concentrations were relatively low (Fig. 3A). Regardless, as compared to the tailings alone, some biochars caused Cd concentrations to increase, while other biochars reduced Cd concentrations (e.g., ARS-Wood, SOR-500, and SOR-700 each applied at 5% by wt., and MIS-700 applied at 1 and 2.5% by wt). Increasing Cd concentrations with biochar application suggests either competitive sorption between Cd and other elements present in the tailings, or these biochars failed to sufficiently raise pH. In the presence of various biochars, Uchimiya et al. (2011) noted that Cd solubility decreased rapidly above pH 8.7; although not measured in the current study, this pH was likely not attained with certain biochars. However, Uchimiya et al. (2011) also noted decreases in Cd availability with other biochars. Similarly, Ippolito et al. (2017) utilized the same extraction technique as in the current study, observing significant decreases (as compared to a control) in bioavailable Cd when lodgepole pine biochar was applied at 5% by wt to four different Cd-contaminated mine tailings. Beesley and Marmiroli (2011) and Fellet et al. (2011, 2014) observed similar Cd reductions.

Bioavailable Cu concentrations, as a function of increasing application rate of various biochars, as compared to tailings alone, are presented in Fig. 3B. Regardless of biochar type, increasing application rates always significantly decreased bioavailable Cu concentration. The least amount of bioavailable Cu was observed when ARS-Wood, SOR-500, and SOR-700 were applied at 5% by wt Ippolito et al. (2017) observed a similar Cu response when lodgepole pine biochar was applied at 5% by wt to mine tailings. Others have also noted decreases in Cu concentrations with biochar application (e.g., Laird et al. 2010; Park et al. 2011; Fellet et al. 2011, 2014).

Bioavailable Mn and Ni concentrations, as a function of increasing application rate of various biochars, as compared to tailings alone, are presented in Fig. 3C, D. In contrast to the sorption and desorption results above, no biochar reduced bioavailable Mn or Ni content as compared to tailings alone. Several biochars led to marked or slight increases in Mn and Ni concentrations, respectively.

Bioavailable Zn concentrations, as a function of increasing application rate of various biochars, as compared to tailings alone, are presented in Fig. 3E. Out of all heavy metals present at the Formosa Mine, Zn bioavailability is likely a major issue with respect to reclamation success, as the SPLP solution contained ~ 8400 mg Zn L^−1^. Out of the selected biochars and rates chosen, only ARS-Wood applied at 5% and MIS-700 applied at 1% reduced bioavailable Zn concentrations to below those of tailings alone. Ippolito et al. (2017) noted a somewhat similar response in bioavailable Zn reduction with lodgepole pine biochar applied at 5% to several Zn-contaminated mine tailings. The authors applied biochar at 10 and 15% (by wt), yet did not observe further decreases in bioavailable Zn concentrations. Previous studies have noted similar responses to the current study (e.g., Ippolito et al. 2019; Kumar et al. 2018; Xu et al. 2017). In the study by Ippolito et al. (2017), the authors utilized a sequential extraction technique, observing that most of the Zn was present in exchangeable and carbonate fractions. Thus, to reduce bioavailable Zn concentrations further, one might consider co-applying ARS-Wood (5%) or MIS-700 (1%) with an appropriate lime source (i.e., a carbonate source) to the Formosa mine tailings. Applying an additional lime source, at a rate to account for mine soil or tailings active and reserve acidity, should help to raise and maintain pH over a longer term, and maintain the presence of Zn as a carbonate mineral precipitate, a less bioavailable form of Zn.

## Conclusion

Biochars have physicochemical properties that make them potential amendments for remediating metal contaminated mine soils and tailings. A three-step procedure was developed to identify biochars that were most effective at reducing heavy metal availability as well as retaining metals. Twenty eight biochars, and a GAC, were screened. Results from the heavy metal sorption step reduced the number of candidate biochars for use in the Formosa tailings remediation from 28 to 16, plus GAC. Results from the heavy metal desorption step reduced the number of biochars from 16 to 10 and eliminated GAC. Results from mixing six of the 10 biochars into Formosa Mine tailings, at various rates, suggested that mixing ARS-Wood biochar applied at 5% or MIS-700 biochar applied at 1% might be the most effective choices for additional Formosa Mine land reclamation studies. Prior to full, on-site use of biochars, this proposed methodology and results from this study could be used to identify specific biochars and application rates to diminish mine soil and tailings metal bioavailability and aid in future remediation of abandoned mine sites globally.

One possible modification to this procedure could include a period of time (e.g., 1 month) for the biochar to react with the tailings under field-moist conditions prior to the 0.01 M CaCl_2_ extraction. This may better demonstrate the ability of the biochars to sorb available metals. Furthermore, as in the case of the Formosa tailings, including lime and other beneficial amendments (e.g., compost or biosolids) in the methodology may demonstrate the utility of multiple interacting amendments to reduce the bioavailability of metal contaminants in tailings at mining impacted sites.

## Figures and Tables

**Table 1:** Biochars utilized for optimal selection for reducing heavy metal bioavailability in a heavy metals contaminated mine tailings

Biochar code	Feedstock	Pyrolysis temperature (°C)
AD-300	*Arundo donax* L. (giant reed)	300
AD-500	*Arundo donax* L. (giant reed)	500
AD-700	*Arundo donax* L. (giant reed)	700
ADF-300	Anaerobically digested fiber	300
ADF-500	Anaerobically digested fiber	500
ADF-700	Anaerobically digested fiber	700
DF-300	*Pseudotsuga menziesii* (Mirb.) Franco (Douglas fir)	300
DF-500	*Pseudotsuga menziesii* (Mirb.) Franco (Douglas fir)	500
DF-700	*Pseudotsuga menziesii* (Mirb.) Franco (Douglas fir)	700
ELY-300	*Elymus glaucus* Buckley (blue wild rye)	300
ELY-500	*Elymus glaucus* Buckley (blue wild rye)	500
ELY-700	*Elymus glaucus* Buckley (blue wild rye)	700
HNS-300	*Corylus avellana* L. shells (Hazelnut shells)	300
HNS-500	*Corylus avellana* L. shells (Hazelnut shells)	500
HNS-700	*Corylus avellana* L. shells (Hazelnut shells)	700
MIS-300	*Miscanthus* (silvergrass)	300
MIS-500	*Miscanthus* (silvergrass)	500
MIS-700	*Miscanthus* (silvergrass)	700
OWO-300	*Quercus garryana* Douglas ex Hook. (Oregon White Oak)	300
OWO-500	*Quercus garryana* Douglas ex Hook (Oregon White Oak)	500
OWO-700	*Quercus garryana* Douglas ex Hook (Oregon White Oak)	700
SBG-300	Spent Brewer’s grain	300
SBG-500	Spent Brewer’s grain	500
SBG-700	Spent Brewer’s grain	700
SOR-300	*Sorghum bicolor* (L.) Moench (sorghum)	300
SOR-500	*Sorghum bicolor* (L.) Moench (sorghum)	500
SOR-700	*Sorghum bicolor* (L.) Moench (sorghum)	700
ARS-Wood	Mixed conifer wood from near Formosa	1100–1400 (via gasification)
GAC	Granulated activated charcoal	Unknown

**Table 2:** Biochar removal (%) of initial SPLP extract containing Cd, Cu, Mn, Ni, and Zn from the Formosa Mine tailings

Biochar code	Cd removal (%)	Cu removal (%)	Mn removal (%)	Ni removal (%)	Zn removal (%)	Total removal (%)
ARS-Wood	*100.00 a*	*100.00 a*	***100.00 a***	*100.00 a*	*100.00 a*	*500.00 a*
MIS-700	*100.00 a*	*99.91 a*	***99.98 a***	*99.99 a*	*100.00 a*	*499.88 a*
ELY-700	*100.00 a*	*99.77 a*	***99.99 a***	*99.76 a*	*99.99 a*	*499.50 ab*
ADF-700	*99.96 a*	*99.94 a*	***99.59 a***	*99.52 ab*	*99.98 a*	*498.98 ab*
SOR-700	*99.89 a*	*99.60 a*	***99.94 a***	*98.93 ab*	*99.92 a*	*498.28 ab*
ELY-500	*100.00 a*	*99.32 a*	***99.65 ab***	*99.12 ab*	*99.99 a*	*498.08 ab*
AD-700	*99.98 a*	*98.98 a*	***99.93 a***	*99.01 ab*	*99.99 a*	*497.89 ab*
ADF-500	*99.93 a*	*99.77 a*	***98.36 abc***	*99.38 ab*	*99.96 a*	*497.39 ab*
MIS-500	*100.00 a*	*99.64 a*	***97.96 abc***	*99.33 ab*	*99.99 a*	*496.92 abc*
SOR-500	*99.82 a*	*98.88 a*	***99.88 a***	*97.85 ab*	*99.88 a*	*496.30 abcd*
AD-500	*99.89 a*	*98.46 a*	***99.73 a***	*98.10 ab*	*100.00 a*	*496.18 abcd*
SOR-300	*99.75 a*	*98.83 a*	***98.27 abc***	*98.48 ab*	*99.60 a*	*494.93 abcd*
GAC	*99.33 ab*	*100.00 a*	***95.16 abcd***	*98.98 ab*	*99.22 a*	*492.69 abcd*
HNS-700	*99.77 a*	*99.78 a*	***91.53 bcdef***	*98.30 ab*	*99.85 a*	***489.23 abcd***
AD-300	*99.74 a*	*99.05 a*	***94.32 abcde***	*96.56 abc*	*99.11 ab*	*488.77 abcd*
ADF-300	*99.64 a*	*99.38 a*	89.79 defg	*98.03 ab*	*99.52 a*	*486.36 abcde*
ELY-300	*98.96 a*	*99.49 a*	91.25 cdef	95.01 bcd	*97.22 abc*	*481.92 abcdef*
OWO-500	*99.85 a*	*99.69 a*	90.48 cdefg	92.06 cde	*95.47 abcd*	477.54 bcdef
OWO-700	*99.70 a*	*99.88 a*	86.88 efghij	91.63 de	*96.81 abc*	474.91 cdefg
MIS-300	*98.25 abc*	*99.35 a*	87.43 defghi	92.57 cde	*96.70 abc*	474.29 defg
HNS-500	*96.26 abcd*	*99.48 a*	84.61 fghijk	90.62 de	93.54 bcde	464.51 efg
OWO-300	93.94 cd	*98.95 a*	88.01 defgh	89.73 e	92.09 cde	462.72 fg
DF-700	91.84 d	*99.86 a*	85.61 fghijk	92.63 cde	90.25 de	460.19 fg
HNS-300	94.88 bcd	*99.30 a*	83.18 ghijk	89.68 e	89.81 e	456.86 g
DF-500	84.58 e	*99.78 a*	79.48 ijk	84.06 f	82.08 f	429.98 h
SBG-700	85.00 e	*98.75 a*	79.90 ijk	82.41 fg	81.92 f	427.99 h
SBG-500	84.76 e	*97.42 a*	80.69 hijk	80.12 fg	83.07 f	426.06 h
SBG-300	82.03 ef	92.58 b	79.21 jk	79.57 fg	81.61 f	415.00 hi
DF-300	79.04 f	86.11 c	78.31 k	79.13 g	78.61 f	401.20 i

The furthest right column is the total removal (i.e., sum of all values within a row), with this column used to rank overall biochar sorption of all heavy metals, from greatest to lowest. Biochar codes are defined in Table 1

Different letterswithin a column signify significant differences between heavy metal removal percent, as determined by Tukey’s range test (*p* < 0.05). For ease of viewing: italic and bolditalic values within a column are statistically similar to one another, corresponding to biochars that sorbed the greatest amount of metal(s)

**Table 3:** The mean (mg kg^−1^) Cd, Cu, Mn, Ni, and Zn desorbed from biochar, after shaking for 24 h in 0.01 M CaCl_2_

Biochar code	Cd desorbed (mg kg^−1^)	Cu desorbed (mg kg^−1^)	Mn desorbed (mg kg^−1^)	Ni desorbed (mg kg^−1^)	Zn desorbed (mg kg^−1^)	Total desorbed (mg kg^−1^)
DF-500	1.52 efg	*0.00 c*	*3.86 lmn*	*0.00 b*	4230 a	4230 a
SBG-500	*0.42 klmn*	*0.00 c*	*1.80 mn*	*0.00 b*	3590 ab	3600 ab
AD-300	1.87 def	*0.00 c*	26.0 efg	*0.00 b*	3370 abc	3400 abc
ELY-300	2.97 ab	*0.00 c*	35.9 de	*0.00 b*	3310 abcd	3350 abc
MIS-500	2.95 ab	*0.00 c*	49.8 c	*0.00 b*	2960 bcde	3010 abcd
DF-700	2.31 cd	*0.00 c*	*8.51 jklmn*	*0.00 b*	2680 bcdef	2690 bcde
HNS-500	3.02 ab	*0.19 c*	14.0 hijkl	*0.00 b*	2630 bcdef	2650 bcde
DF-300	*0.00 n*	3.02 b	*0.00 n*	*0.00 b*	2550 bcdef	2560 bcde
SBG-700	0.80 hijk	*0.00 c*	*1.85 mn*	*0.00 b*	2330 cdefg	2330 cdef
OWO-700	1.78 def	*0.00 c*	23.8 fghi	0.57 a	2220 cdefg	2250 cdef
HNS-300	3.16 ab	*0.00 c*	23.4 fghi	*0.00 b*	2140 cdefg	2170 cdef
MIS-300	3.36 a	*0.00 c*	37.0 de	*0.00 b*	2100 defg	2150 cdef
AD-500	0.88 hijk	*0.00 c*	19.2 fghij	*0.00 b*	1960 efg	1980 def
GAC	2.17 cd	*0.00 c*	35.7 de	*0.00 b*	1890 efg	1920 def
OWO-300	1.95 de	3.95 a	*7.40 klmn*	*0.00 b*	1880 efg	1890 defg
SBG-300	*0.47 jklmn*	*0.20 c*	*3.44 lmn*	*0.00 b*	1770 efgh	1770 defg
OWO-500	1.06 ghij	*0.00 c*	26.7 efg	*0.00 b*	1730 efgh	1760 defg
HNS-700	2.75 bc	*0.00 c*	25.5 efgh	*0.00 b*	1640 fgh	1660 efg
ADF-500	1.12 ghi	*0.00 c*	30.5 def	*0.00 b*	*1240 ghi*	1270 fgh
ADF-300	1.49 efg	*0.00 c*	105 a	*0.00 b*	*1100 ghi*	*1210 fghi*
MIS-700	1.31 fgh	*0.00 c*	40.9 cd	*0.00 b*	*611 hi*	*653 ghi*
SOR-300	0.67 ijklm	*0.00 c*	68.2 b	*0.00 b*	*583 hi*	*652 ghi*
ELY-500	0.66 ijklm	*0.00 c*	26.5 efg	*0.00 b*	*346 i*	*374 hi*
ADF-700	0.72 hijkl	*0.00 c*	28.1 ef	*0.00 b*	*174 i*	*203 hi*
AD-700	*0.09 mn*	*0.00 c*	*10.5 jklmn*	*0.00 b*	*39.1 i*	*49.7 hi*
ELY-700	*0.19 lmn*	*0.00 c*	13.1 ijklm	*0.00 b*	*32.6 i*	*45.8 hi*
ARS-Wood	*0.10 mn*	*0.00 c*	16.0 ghijk	*0.00 b*	*6.65 i*	*22.7 hi*
SOR-500	*0.00 n*	*0.00 c*	*0.88 n*	*0.00 b*	*9.91 i*	*10.8 i*
SOR-700	*0.00 n*	*0.00 c*	*0.00 n*	*0.00 b*	*5.90 i*	*5.90 i*

The furthest right column is the total amount of heavy metals desorbed (i.e., the row sum), with this column used to rank overall biochar desorption of all heavy metals, from greatest to least. Biochar codes are defined in Table 1.

Different letters within a column signify significant differences between heavy metal desorption, as determined by Tukey’s range test (*p* < 0.05). For ease of viewing: italicized values within a column are statistically similar to one another, corresponding to biochars that desorbed the least amount of metal(s)

## Data Availability

The datasets used or analyzed during the current study are available from the corresponding author on reasonable request.
